# Photodegradation of polyethylene debris in water by sulfur-doped TiO_2_: system optimization, degradation mechanism, and reusability

**DOI:** 10.1007/s11356-023-31460-1

**Published:** 2023-12-14

**Authors:** Ahmed Sharara, Mahmoud Samy, Mohamed Mossad, Mohamed Gar Alalm

**Affiliations:** Public Works Engineering Department, Faculty of Engineering, Mansoura University, Mansoura, 35516 Egypt

**Keywords:** Degradation mechanism, Mass loss, Microplastics, Optimum parameters, Polyethylene bags, Photodegradation, Reactive radicals

## Abstract

Given the immense threats of microplastics, we herein investigate photodegrading the debris of polyethylene bags (PBs) by sulfur-doped titanium dioxide. The optimization of operating parameters showed that controlling the water pH at 3 and introducing PBs by 0.10 g/L under a catalyst dose of 1.25 g/L reduced the polyethylene mass by 3.10% in 7 h, whereas raising the catalyst dose to 3 g/L improved the mass reduction to 4.72%. The extension of degradation time to 100 h at pH 3, catalyst dosage of 3 g/L, and PBs concentration of 0.10 g/L increased the mass loss ratio to 21.74%. Scanning electron microscopy of PBs after 100 h of photodegradation showed cracks on the surface accompanied by the increase of carbonyl index from 0.52 to 1.41 confirming the breakdown of the polymeric chain. Total organic carbon increased from 0.80 to 7.76 mg/L in the first 10 h of photodegradation, then decreased to 1 mg/L after extending the reaction time to 100 h due to the mineralization of organic intermediates generated from the photodegradation of PBs. Trapping tests exhibited the major role of hydroxyl radicals in the degradation system, and the catalyst showed high stability under five repetitive runs. This study proposes an efficient treatment system that can be implemented on a wider scale utilizing the synthesized catalyst to degrade plastics efficiently before their release to water streams.

**Supplementary Information:**

The online version contains supplementary material available at 10.1007/s11356-023-31460-1.

## Introduction

Due to the large-scale production of plastic, plastic waste can be detected in the environment which has lured global worries due to the detrimental effects of plastic waste on the ecosystem (Ariza-tarazona et al. 2020). Plastic items can remain in the environment for long periods due to their low biodegradability (Geyer et al. 2017). Thus, large items can be broken into smaller plastic particles such as mesoplastics (< 2.50 cm), microplastics (< 5 mm), and nanoplastics (< 1 µm) via the environmental degradation processes (e.g., abrasion, hydrolysis, oxidation, UV radiation, microbial decomposition) (Hamd et al. 2022). The annual production of plastic is over 380 million tons, and it is expected to reach 2 billion tons by 2050 (Paço et al. 2017; Janaswamy et al. 2022). Around 79% of plastic and its fragments could be disposed of in landfills which is harmful to bacteria, plants, and humans due to the leaching of these plastics into the soil and groundwater (Ahmad et al. 2023). Plants can uptake plastic, especially with smaller size which could lead to the accumulation of these compounds in the food chain (Ahmad et al. 2023). Further, plastic’s surface can act as a carrier for toxic pollutants which may lead to the transfer of these pollutants to the food web (Tang et al. 2020). Further, greenhouse gases can be produced due to the accumulation of plastics in landfills (Tang et al. 2020). On the other hand, plastic waste can be managed by incineration which results in the production of greenhouse gases (e.g., CO_2_) and the increase of risks related to global warming leading to the environmental pollution (Ahmad et al. 2023).

On the other hand, plastic fragments (meso, micro, and nanoplastics) can exist in aquatic life via the release of treated effluents from wastewater treatment plants (WWTPs), as WWTPs could not efficiently remove plastic fragments (Enfrin et al. 2020). For instance, over 4 million microplastics (MPs) can be released daily from each WWTP into water streams in the USA (Mason et al. 2016). Further, plastic can exist in seas, oceans, and lakes due to the direct disposal of plastic in water streams or/and owing to the natural transfer of plastic to water streams due to their low density (Jiang et al. 2018). Plastic and its fragments can migrate long distances in the aquatic environment by the marine currents which could damage aquatic life (Ariza-tarazona et al. 2019). Aquatic organisms can ingest plastic and its fragments which may result in the blockage of the digestive tract followed by death (Eerkes-medrano et al. 2015; Ateia et al. 2022). Additionally, the consumption of plastic by marine organisms can contribute to the accumulation of plastic in the human body via the food chain (Ariza-tarazona et al. 2020). According to recent reports, 5 g of plastic fragments can enter human body every week (Ariza-tarazona et al. 2020). The impacts of plastic and its fragments on human health are still mysterious. Howbeit, some studies reported that plastics could cause physical damage and biological stress in humans (Revel et al. 2018; Dong et al. 2020). Therefore, tertiary treatment unit has to be added to conventional WWTPs to efficiently and completely degrade plastic fragments prior the release to water streams as well as strict measures have to be executed to prevent the direct release of plastics into the aquatic life.

Biological, physical, and chemical techniques are not efficient for the degradation of plastic and its fragments due to the produced sludge, long treatment time, production of secondary microplastics (MPs), and high chemical consumption (Dey et al. 2021). Recently, advanced oxidation processes have shown remarkable effectiveness in the degradation of plastics and their fragments such as photocatalysis, Fenton, and ozonation processes (Li et al. 2022; Liu et al. 2022; Ortiz et al. 2022). Fenton and ozonation processes suffer from some demerits such as the use of hazards and expensive chemicals as well as the generation of large volumes of sludge in the case of Fenton process which obstruct the full-scale application of the aforementioned processes (Hamd et al. 2022). Photocatalysis, an advanced oxidation process, is featured by its high degradation performance, ability to utilize solar light, and green nature (Ariza-tarazona et al. 2020). In photocatalysis process, the degradation of plastic and its fragments can take place via the generated reactive species resulting in breaking polymer chain in plastics followed by forming short chain intermediaries and mineralization to water and carbon dioxide (Lu et al. 2021). Nonetheless, the photocatalysis process suffers from some defects such as the wide bandgap and the fast reunite between electrons and holes in the traditional catalysts such as TiO_2_ and ZnO which suppressed the accelerated degradation (Cui et al. 2020). To overcome the above-mentioned issues, bare photocatalysts have been modified by doping with metals or/and non-metals (Murillo-Sierra et al. 2018; Karim and Shriwastav 2020). Zinc et al. (2019) employed platinum/zinc oxide nanorod for the photodegradation of polyethylene film (1 cm × 1 cm) in 175 h using a halogen lamp (Zinc et al. 2019). They reported an increase in the carbonyl index by 2.10 times in the case of doped-ZnO compared to pure ZnO (Zinc et al. 2019). Llorente-García et al. (2020) degraded microplastics (3 mm × 3 mm and 5 mm × 5 mm) by N-TiO_2_ under UV irradiation for 50 h at pH 3 (Llorente-García et al. 2020). The mass loss ratios were 1.38% and 0.97% in the case of microplastics with 3 mm × 3 mm and 5 mm × 5 mm, respectively indicating that the increase of plastic’s size could decrease the degradation rate. Herein, an efficient photocatalyst (sulfur-doped TiO_2_ (S-TiO_2_)) that was previously employed in our previous studies for the degradation of emerging pollutants such as trimethoprim and 2,4-dichlorophenol was utilized for the degradation of polyethylene bags (Samy et al. 2023b, a). Doping with sulfur reduced the bandgap to 2.85 eV which improved the absorption in the visible light region as well as sulfur ions could trap electrons inhibiting the recombination between charge carriers, thereby enhancing the photocatalytic performance and the generation of reactive species. The efficient degradation of plastic waste using the fabricated photocatalyst can participate in reducing the reliance on landfilling and incineration and decreasing the release of plastic waste to water streams which decrease the aforementioned environmental hazards related to plastic waste.

In this study, polyethylene bags (PBs) were collected and cut into 1 cm × 1 cm pieces, and the photocatalytic degradation of PBs was performed using S-TiO_2_. The morphological changes as well as the alterations on PBs surface were investigated as a result of the photodegradation process using scanning electron microscopy and Fourier transform infrared spectroscopy. Further, the degradation of PBs was monitored under different operating parameters such as pH, PBs concentration, reaction time, and catalyst dose. Additionally, the degradation mechanism of PBs was investigated as well as the recyclability performance of the photocatalyst was assessed.

## Materials and methods

### Materials

p-Benzoquinone (C_6_H_4_O_2_, 99%), tert-butyl alcohol (C_4_H_10_O, 99%), and ammonium oxalate (C_2_H_8_N_2_O_4_, 99%) were purchased from Sigma-Aldrich.

### Experimental procedures

The preparation of S-TiO_2_ was conducted as previously described in the previous study (Gar Alalm et al. 2018). Polyethylene bags (PBs) were compiled from the household garbage of a residential area, washed with distilled water, left for drying at room temperature, and cut into small pieces (1 cm × 1 cm). The collected PBs are characterized by their low density and transparency. The degradation of PBs was attained in a photoreactor using a 400 W metal halide lamp as a light source, a 250 mL beaker that contains 100 mL of distilled water, and a magnetic stirrer for achieving excellent contact between plastic pieces and catalyst particles. The spectra of the lamp were provided in the supplementary file (Fig. S1). The distance between the lamp and water surface was 10 cm with an intensity of 45,000 lux. Initially, control experiments were performed using light only, catalyst only, light with catalyst, and PBs without light and catalyst. The effect of light intensity was tested by changing the distance between the lamp and the solution surface (10–40 cm) to specify the intensity that could attain the highest degradation. The light intensity was measured by a lux meter (Milwaukee SM700). Further, the effect of pH (2–9), PBs concentration (0.10–0.30 g/L), and catalyst dosage (0.25–5 g/L) was studied. The experiments were conducted at room temperature 30 °C, whereas the temperature increased to 40 °C during the reaction as a result of the heat emitted by the lamp.

The optimization of operating parameters was performed by varying their values as shown in Table 1. Twenty experiments were selected to attain interaction between the operating parameters based on the response surface method (RSM) coupled with central composite design (CCD). A polynomial model was developed to relate the mass loss ratio of PBs after 7 h with the independent parameters such as pH, catalyst dose, and PBs concentration. The suitability of the model to predict the mass loss ratio was examined by performing analysis of variance (ANOVA). According to the developed model, the decrease of pH values to 2 and 3 and the raising of catalyst dosage to 3 and 5 g/L as well as the extension of reaction time to 100 h were performed to enhance the degradation efficacy. The effectiveness of the synthesized catalyst was validated via measuring the comparison factor (CF) in our study (Eq. (1)), then our comparison factor was compared with other studies.

1 $${\text{CF}}=\left(\mathrm{Efficiency }\times \mathrm{ PBs\;concentration}\right)/\left(\mathrm{Photocatalyst\;dose}\times {\text{Time}}\right)$$

where efficiency is the mass loss ratio, PBs concentration and photocatalyst dose are in g/L, and the time is in hours.

**Table 1:** Values of studied operating parameters

Independent variables	Units	Levels				
		−2	−1	0	1	2
PBs concentration	g/L	0.10	0.15	0.20	0.25	0.30
pH	−	5	6	7	8	9
Catalyst dose	g/L	0.25	0.50	0.75	1	1.25

The primary reactive species were specified using trapping experiments in which tert-butyl alcohol, benzoquinone, and ammonium oxalate were employed as quenchers of hydroxyl radicals, superoxide radicals, and holes, respectively. The quenchers’ concentrations were 50 mM. Also, in the recyclability test, the solution was left 1 h to allow the catalyst particles to settle after each cycle, then the supernatant was decanted. Subsequently, the catalyst particles were compiled from the bottom of the reactor after drying for reusing the catalyst particles in succeeding cycles.

### Analytical methods

The morphology and particle size of the fabricated S-TiO_2_ were explored by a transmission electron microscopy (TEM), and the crystallinity of S-TiO_2_ was investigated using an X-ray diffraction spectroscopy (XRD). The chemical elements of the prepared S-TiO_2_ were determined using an energy-dispersive X-ray (EDX) spectroscopy. Further, the bandgap of S-TiO_2_ was estimated via a UV-Vis spectrophotometer via Kubelka-Munk equation by plotting photon energy (hv) versus (F(R)hv)^0.5^, where F(R) means the function of reflectance (Ariza-tarazona et al. 2020). Moreover, the separation of electrons and holes was evaluated using fluorescence spectrophotometer by recording the photoluminescence (PL) spectra. Regarding the PBs, scanning electron microscopy (SEM) was performed before and after the degradation to evaluate the morphological changes. Additionally, Fourier transform infrared spectroscopy (FTIR) was conducted before and after degradation to investigate the changes in surface functional groups and confirm the degradation of PBs based on carbonyl index (CI). CI is equal to the proportion of carbonyl peak (1550–1810 cm^−1^) to reference peak (1368 cm^−1^) (Ortiz et al. 2022). CI is an index of the degradation of PBs, where the increase of CI means the efficient degradation of plastics. FTIR spectra were recorded between 4000 and 400 cm^−1^ with a spectral resolution of 4 cm^−1^. The models of the aforementioned equipment were mentioned in our previous studies (Samy et al. 2023b, a).

After the degradation process, the treated solution was filtered to collect PBs followed by drying at room temperature to measure the mass loss ratio using a sensitive balance as shown in Eq. (2). Additionally, the mineralization of PBs was explored using total organic carbon analyzer (TOC, Shimadzu).

2 $${\text{ML}}\left(\mathrm{\%}\right)=\left({{\text{M}}}_{0}-{{\text{M}}}_{{\text{t}}}\right)/{{\text{M}}}_{0}\times 100\mathrm{\%}$$

where ML is the mass loss ratio (%) of PBs, M_0_ is the initial mass of PBs (g), and M_t_ is the mass of PBs at the end of reaction (g).

## Results and discussion

### Characterizations of S-TiO_2_ and PBs

**Fig. 1:**
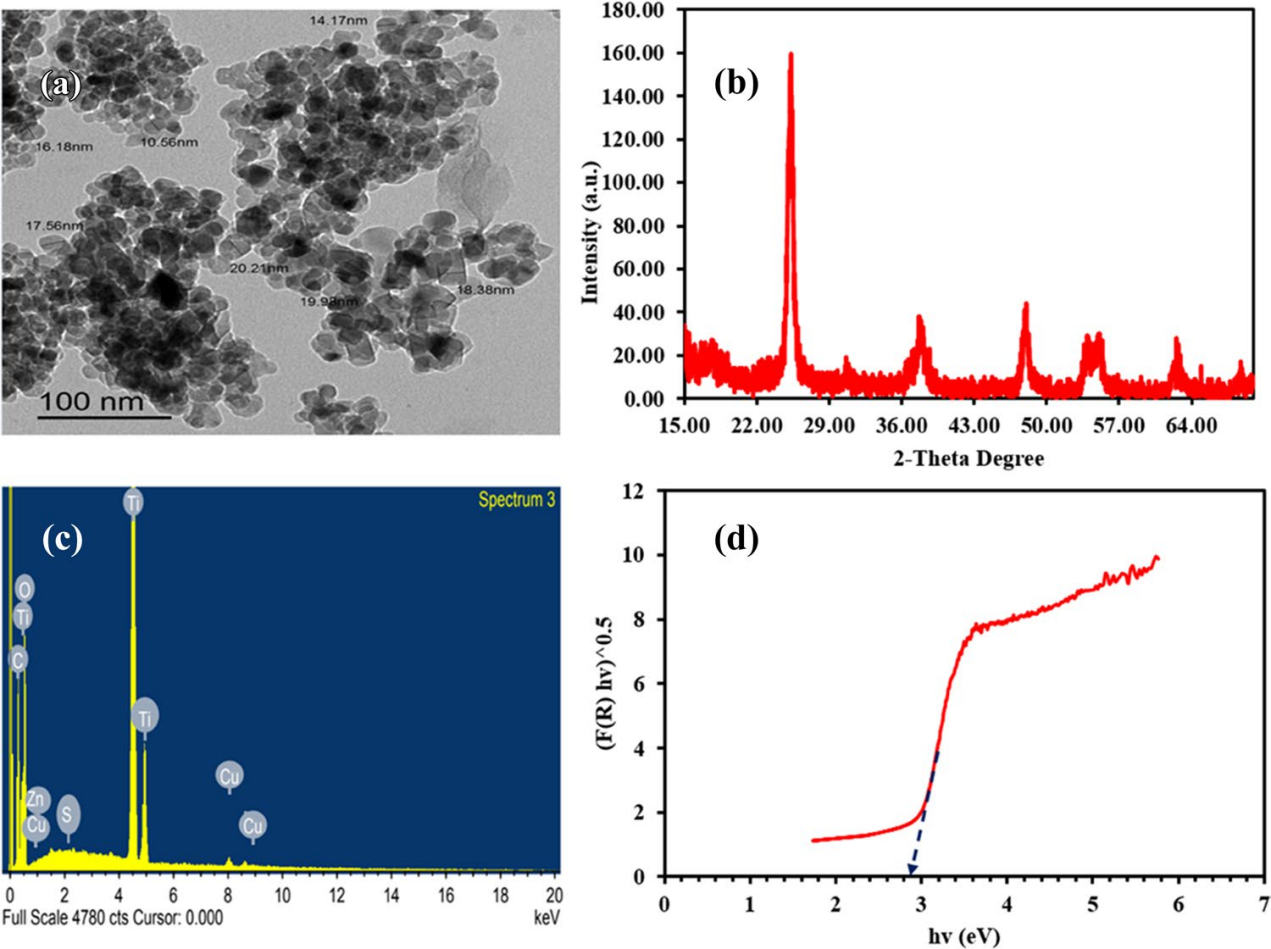
**a** TEM image, **b** XRD pattern, **c** EDX pattern, and **d** bandgap of the prepared S-TiO_2_

The TEM image in Fig. 1a shows that the particles have spherical-like morphology with a particle size range (10–21 nm). The XRD pattern in Fig. 1b depicts a main peak at 25.3° which is indexed to anatase TiO_2_. Further, the peaks at nearly 38°, 48°, 53.9°, 55.4°, and 62.7° are imputed to the anatase TiO_2_ (JCPDS no. 21-1272) (Ramos-Delgado et al. 2013). The sulfur in the doped TiO_2_ did not show any unique peaks due to its low content as well as the regular distribution of its ions with anatase crystallite (Hamadanian et al. 2009). EDX pattern in Fig. 1c depicts the growth of Ti, O, and S confirming the chemical composition of S-TiO_2_. Figure 1d shows the bandgap of the synthesized S-TiO_2_ using the function of reflectance (F(R)). F(R) was measured as described in our previous work (Samy et al. 2020b). The bandgap was 2.85 eV which is lower than that of pure TiO_2_ (3.20 eV) affirming the role of sulfur ions in narrowing the bandgap and boosting the utilization of broad area of the solar light, thereby improving the generation of reactive radicals. Figure S2 shows the PL spectra of pure TiO_2_ and S-TiO_2_ with a maximum emission at nearly 385 nm. The intensity in the case of S-TiO_2_ was lower than that of TiO_2_ confirming the improvement of the separation of charge carriers in the case of doped TiO_2_.

**Fig. 2:**
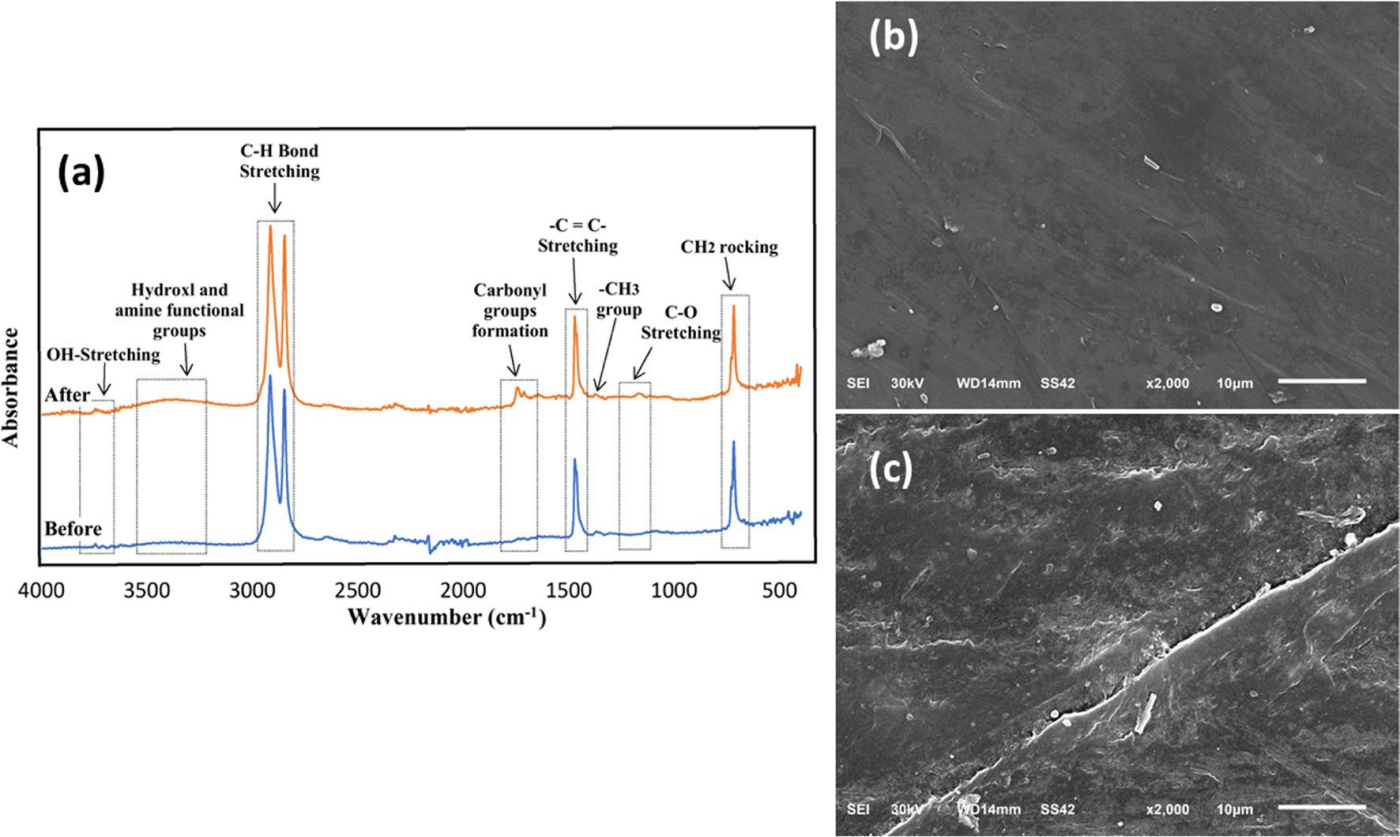
**a** FTIR spectra of PBs before and after 100 h of the photodegradation and SEM images of PBs **b** before degradation and **c** after degradation

Figure 2a shows the FTIR spectra of PBs before and after the photodegradation. The comparison between the FTIR spectra of PBs before and after degradation clearly shows significant changes in the chemical structure after the photodegradation by S-TiO_2_. The observed peaks at 2915, 2848, 1471, and 719 cm^−1^ are due to the vibrations of the alkyl groups in the plastic (Ariza-Tarazona et al. 2020). Specifically, the peaks at 2915 and 2848 cm^−1^ are imputed to C–H bond stretching vibrations, while the peaks at 1471 and 719 cm^−1^ are indexed to –C=C– bond stretching and CH_2_ rocking deformation, respectively. A peak appeared at 1368 cm^−1^, indicating a slight deformation of the –CH_3_ group, which remained relatively stable after irradiation (Tofa et al. 2019b; Ariza-Tarazona et al. 2020). In addition, notable changes were observed in the range of 1650 to 1750 cm^−1^, where significant peaks associated with carbonyl groups (C=O bonds) appeared in the case of PBs after degradation. This suggests the increase of carbonyl functional groups during the photocatalytic degradation process.

The observed changes in the FTIR spectrum confirm the successful breakdown of the plastic by the S-TiO_2_ photocatalyst. To further evaluate the extent of degradation, the carbonyl index (CI) was calculated, which is an indicator of the degradation of plastic. By comparing the absorbance of the carbonyl peak (1743 cm^−1^) with the reference peak (1368 cm^−1^), the CI before irradiation was determined to be 0.52, indicating a relatively low concentration of carbonyl functional groups. However, after irradiation, the CI increased to 1.41, indicating a higher concentration of carbonyl groups resulting from the breakdown of polymer chains and the formation of new carbonyl groups.

Figure 2b, c shows the SEM micrographs of PBs before and after the degradation for 100 h. Figure 2b demonstrates a smooth and homogenous surface of PBs before degradation. In the case of PBs after the degradation as given in Fig. 2c, the surface becomes rougher as well as cracks or fractures appear on the surface which affirmed the breakdown of polymer chains in PBs as a result of the degradation.

### Control experiments

**Fig. 3:**
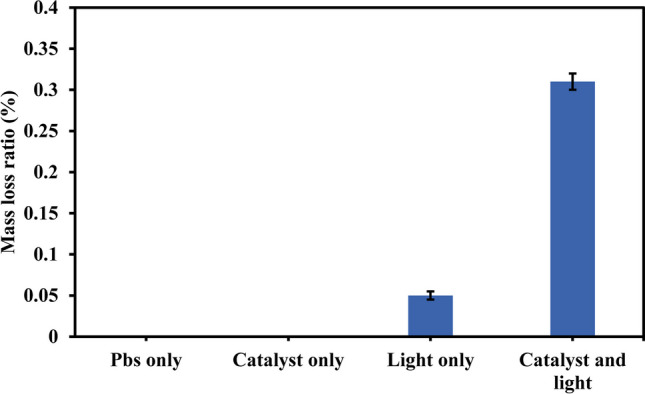
Control experiments of PBs degradation

Figure 3 shows the mass loss ratios of PBs in the case of using light only without catalyst, catalyst only without light, catalyst with light, and PBs without light and catalyst at pH 7, catalyst dose of 0.25 g/L, PBs concentration of 0.20 g/L, light intensity of 45000 lux, and reaction time of 7 h. In the case of PBs degradation without catalyst and light, there was no change in the mass of PBs after mixing for 7 h. Further, in the case of light only, the mass loss ratio was only 0.05%. The limited mass loss in the case of photolysis and PBs without catalyst and light affirmed the stability of polyethylene bags under natural environmental conditions. In the case of catalyst only, no change in mass was observed, as the degradation of PBs by the catalyst could not take place without illumination (Ariza-tarazona et al. 2019). On the other hand, the mass loss ratio increased to 0.31% after 7 h due to the illumination of catalyst which resulted in the production of reactive oxygen species, thereby degrading PBs polymer chain (Ariza-tarazona et al. 2020). The obtained mass loss ratio was attained under low catalyst dose, short reaction time, and large plastic size compared to previous studies. Ariza-Tarazona et al. (2020) reported a mass loss ratio of 0.15% at a temperature of 20 °C, nitrogen-doped TiO_2_ dose of 4 g/L, microplastic concentration of 4 g/L, and reaction time of 50 h (Ariza-tarazona et al. 2020). Figure S3 shows the effect of light intensity in the presence of the catalyst (0.25 g/L) on the mass loss ratio. The intensities were 45,000, 30,000, 25,000, and 20,000 lux corresponding to the distances between the lamp and water surface of 10, 20, 30, and 40 cm, respectively. The highest mass loss ratio was attained at a light intensity of 45,000 lux (0.31%), as the increase of light intensity could enhance the generation of reactive radicals (Gogoi et al. 2022). Thus, all the next experiments were performed at this light intensity. In the next experiments, the optimization of operating parameters such as pH, catalyst dose, and PBs concentration was performed to achieve higher mass loss ratios.

### Optimization of operating parameters

A polynomial model shown in Eq. (3) with *R*^2^ of 0.97 was developed to link the mass loss ratio with the independent parameters (pH, catalyst dose, and plastic concentration) using Minitab@20. Table 2 shows the experimental mass loss ratios and the expected mass ratios using the model under different operating parameters. Additionally, analysis of variance (ANOVA) in Table 3 affirms the convenience of the model due to the low *P* and high *F* values.

3 $$\mathrm{Mass\;loss\;ratio }\left(\mathrm{\%}\right)=3.33-3.85\mathrm{ X}+3.419\mathrm{ Y}-0.764\mathrm{ Z}+12.68 {{\text{X}}}^{2}-0.433 {{\text{Y}}}^{2}+0.054 {{\text{Z}}}^{2}-3.80\mathrm{ X Y}-0.30\mathrm{ X Z}-0.16\mathrm{ Y Z}$$

where X, Y, and Z are PBs concentration (g/L), catalyst dose (g/L), and pH, respectively.

**Table 2:** Expected and actual mass loss ratios under different operating conditions

Run	Coded values of parameters		Actual values of parameters			Mass loss ratio		
	PBs conc.	Catalyst dose	pH	PBs conc.	Catalyst dose	pH	Actual	Expected
1	1	1	1	0.25	1	8	0.8	0.66
2	1	1	−1	0.25	1	6	1.2	1.146
3	1	−1	1	0.25	0.50	8	0.4	0.39025
4	1	−1	−1	0.25	0.50	6	0.8	0.71625
5	−1	1	1	0.15	1	8	1.2	1.1578
6	−1	1	−1	0.15	1	6	1.7	1.5838
7	−1	−1	1	0.15	0.50	8	0.77	0.69805
8	−1	−1	−1	0.15	0.50	6	0.95	0.96405
9	−2	0	0	0.10	0.75	7	1.33	1.3554875
10	2	0	0	0.30	0.75	7	0.55	0.6098875
11	0	2	0	0.20	1.25	7	1.1	1.1923875
12	0	−2	0	0.20	0.25	7	0.31	0.3028875
13	0	0	2	0.20	0.75	9	0.66	0.6958875
14	0	0	−2	0.20	0.75	5	1.4	1.4478875
15	0	0	0	0.20	0.75	7	0.85	0.8558875
16	0	0	0	0.20	0.75	7	0.845	0.8558875
17	0	0	0	0.20	0.75	7	0.844	0.8558875
18	0	0	0	0.20	0.75	7	0.851	0.8558875
19	0	0	0	0.20	0.75	7	0.853	0.8558875
20	0	0	0	0.20	0.75	7	0.847	0.8558875

**Table 3:** ANOVA for the mass loss ratio of PBs

Source	DF	Sum of squares	Mean square	***F*** value	***P*** value
Model	9	2.06034	0.228926	35.29	0
Linear	3	1.89473	0.631575	97.37	0
X	1	0.55503	0.555025	85.57	0
Y	1	0.7921	0.7921	122.12	0
Z	1	0.5476	0.5476	84.42	0
Square	3	0.13296	0.04432	6.83	0.009
X*X	1	0.02527	0.025273	3.9	0.077
Y*Y	1	0.01839	0.018391	2.84	0.123
Z*Z	1	0.07387	0.073873	11.39	0.007
2-way interaction	3	0.03265	0.010883	1.68	0.234
X*Y	1	0.01805	0.01805	2.78	0.126
X*Z	1	0.0018	0.0018	0.28	0.61
Y*Z	1	0.0128	0.0128	1.97	0.19
Error	10	0.06486	0.006486		
Total	19	2.1252			

**Fig. 4:**
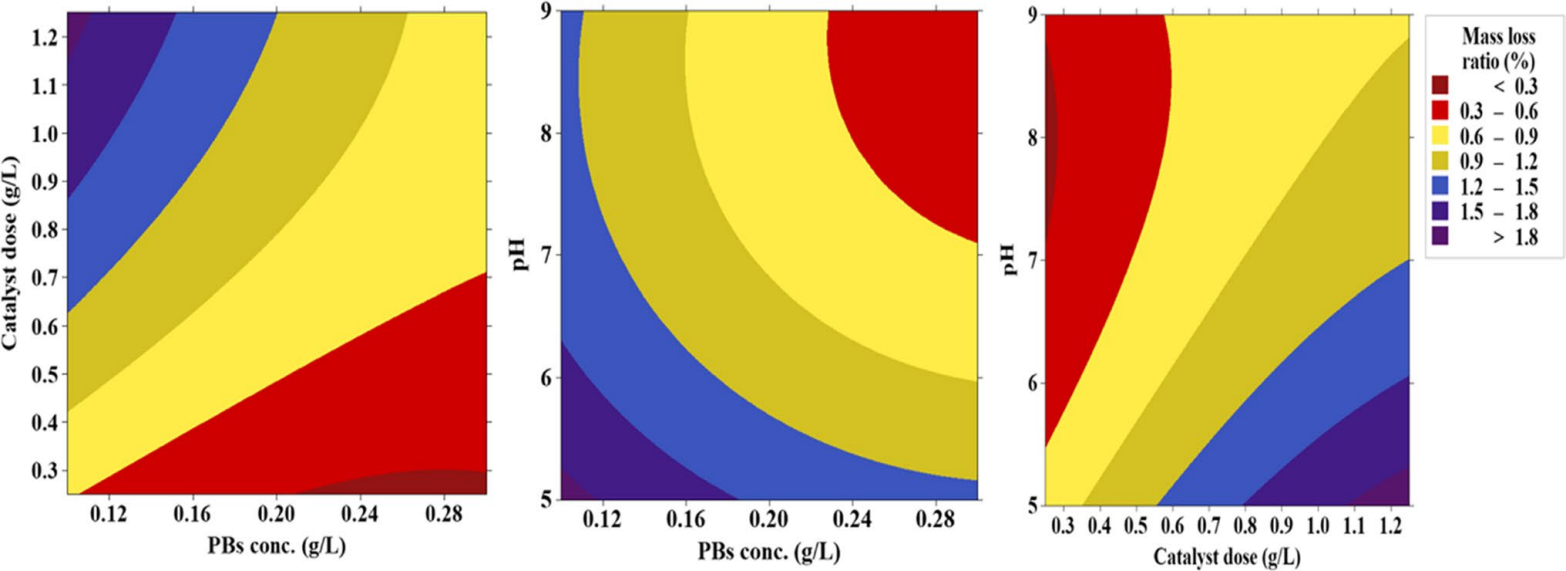
Effects of operating parameters on mass loss ratios

Figure 4 shows the effects of operating parameters on mass loss ratios. The results indicated that the increase of catalyst dose enhanced the degradation of polymeric chain; therefore, the optimal catalyst dose was 1.25 g/L. Elevating the catalyst dose could contribute to the generation of more hydroxyl radicals that would attack the weak points in the polymeric chain resulting in the generation of polyethylene alkyl radicals (Kriston 2010). Further, peroxy radicals could be formed via reacting alkyl radicals with oxygen that might abstract H from another polymeric chain forming hydroperoxide (Ariza-tarazona et al. 2020). Subsequently, hydroperoxide could be disassociated due to the weak O–O bond into two radicals (oxy and hydroxyl radicals) that could abstract unstable hydrogens from other polymeric chains (Tofa et al. 2019a). Therefore, the raising of the produced hydroperoxide as a result of the increase of catalyst dose could participate in accelerating the polymeric chain decomposition.

The effect of pH was also explored by varying pH (5–9) stating that pH 5 was the optimum. In the case of acidic conditions, H^+^ ions would be available which could accelerate the formation of hydroperoxide leading to the elevation of oxy and hydroxyl radicals with high reactivity than peroxy radicals, thereby improving plastic degradation (Kriston 2010). Further, the zeta potential of TiO_2_ is high at low pH which suppressed the agglomeration of catalyst particles (Mikulášek et al. 1997). The deagglomeration of catalyst particles could contribute to the increase of active surface area and the interaction between TiO_2_ nanoparticles and PBs resulting in the improvement of degradation efficacy. On the other hand, at high pH values, the mass loss ratio decreased might be due to the possible agglomeration of catalyst particles leading to the decline of active sites and the decrease of interaction between the catalyst particles and PBs. The point of zero charge of S-TiO_2_ is 6.25 as reported by Jafari et al. (2020), so the catalyst’s charge is positive at acidic condition (pH < 6.25) and negative at alkaline condition (pH > 6.25) (Jafari et al. 2020). On the other hand, the surface charge of plastics (e.g., PBs) can be negative due to the environmental weathering (Fotopoulou and Karapanagioti 2012; Liu et al. 2020b). Thus, the degradation performance was higher at acidic condition due to the attractive forces between the positively charged S-TiO_2_ and negatively charged PBs, whereas the mass loss ratio decreased at high pH values owing to the repulsive forces.

Regarding the effect of PBs concentration, the raising of PBs concentration above 0.10 g/L could contribute to the decrease of the mass loss ratio. The increase of PBs concentration requires more radicals and a longer time, so the mass loss of PBs would be higher at lower PBs concentrations compared to higher concentrations at the same reaction time. Table 4 shows the optimum operating parameters and the expected mass loss ratio under the optimum value.

**Table 4:** Optimum operating parameters for degrading PBs and the actual and expected mass loss ratios under the optimum values

Parameters	Optimum values
PBs concentration (g/L)	0.10
pH	5
Catalyst dose (g/L)	1.25
Expected mass loss ratio under 7 h	2.58
Experimental mass ratio under 7 h	2.70

To check the appropriateness of the model to predict the mass loss ratio, the model’s validity was assessed by experimenting with the optimum condition as shown in Table 4, and the mass loss ratio was compared with the value obtained from the model under the optimum condition. The difference between the expected and experimental mass loss ratio under the optimum condition was low which affirmed the applicability of the model.

### Improvement of the mass loss ratio

**Fig. 5:**
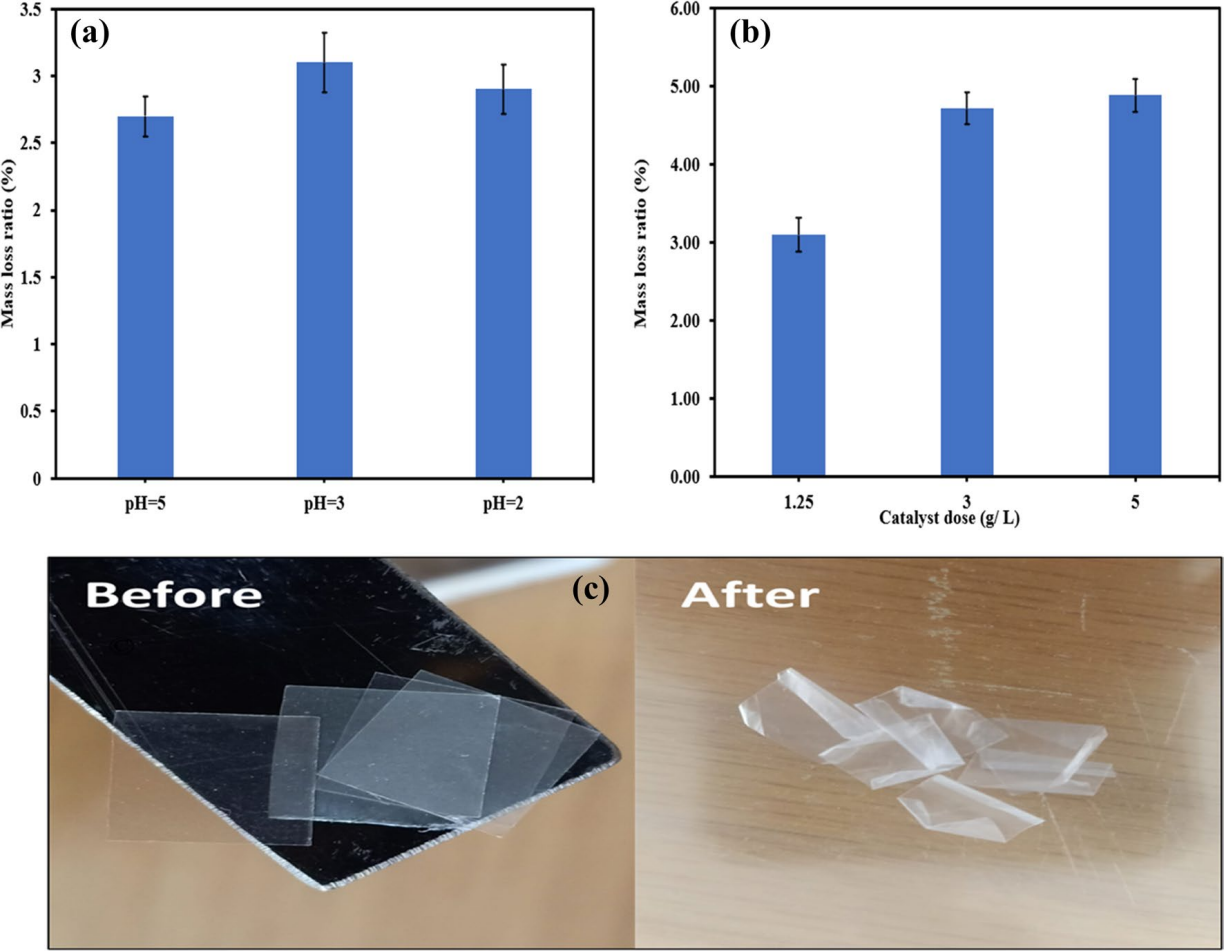
Effect of **a** pH and **b** catalyst dose on the degradation of PBs and **c** PBs before and after the photodegradation

Despite the high stability of PBs, especially with their large size (1 cm × 1 cm), we continued our endeavors to improve the degradation efficiency. Thus, decreasing pH, increasing catalyst dose, and prolonging the reaction time were performed. The mass loss ratio increased from 2.70% under the optimal condition (pH 5, catalyst dose of 1.25 g/L, and PBs concentration of 0.10 g/L) to 3.10% after decreasing pH value to 3 due to the abundance of H^+^ ions that could drive the reaction towards the generation of hydroperoxide which enhanced the degradation of PBs as shown in Fig. 5a (Tofa et al. 2019a). Howbeit, the mass loss percentage was only 2.90% at pH 2. The declined mass loss in the case of pH 2 compared to pH 3 could be owing to the quenching nature of H^+^ ions towards the generated hydroxyl radicals at very low pH values as described in Eq. (4). Radwan et al. (2018) reported the same conclusion during the removal of phenol in an electro-Fenton system (Radwan et al. 2018).

4 $${}^{\bullet }{\text{OH}}+{{\text{H}}}^{+}+{{\text{e}}}^{-}\to {{\text{H}}}_{2}{\text{O}}$$

On the other hand, increasing the catalyst dose to 3 g/L at pH 3 and PBs concentration of 0.10 g/L improved the mass loss ratio to 4.72% due to the increase of the generated radicals that could abstract more hydrogens from the polymeric chain, thereby improving the decomposition performance as depicted in Fig. 5b. However, the increase of catalyst dose to 5 g/L showed a mass loss ratio of 4.89%. The raising of catalyst dosage did not frequently enhance the degradation performance due to the tendency of catalyst particles to agglomerate at higher doses which decreased the number of active sites (Liu et al. 2020a). Also, the increase of catalyst dose might result in scattering the photons which could deprive many active sites from illumination resulting in the decline of the degradation efficiency (Samy et al. 2020a, 2021a).

To increase the mass loss ratio and check the stability of the photocatalyst under long reaction time, the degradation of PBs was conducted over 100 h under pH 3, catalyst dosage of 3 g/L, and PBs concentration of 0.10 g/L. The mass loss ratio jumped to 21.74% after the long reaction time because of the continuous generation of reactive radicals that could attack the polymeric chain (Ariza-tarazona et al. 2019). The improvement of mass loss ratio with time affirmed the stability of the catalyst and its potential to generate reactive species under long operational time. Figure 5c demonstrates the changes in PBs before and after photodegradation for 100 h.

**Fig. 6:**
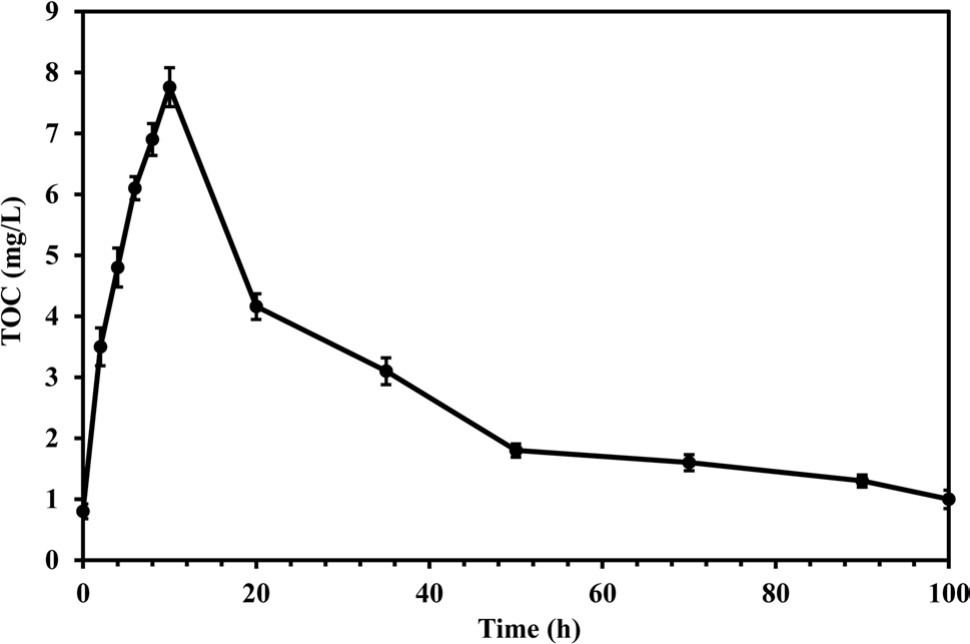
Mineralization of PBs over 100 h

On the other hand, total organic carbon (TOC) was monitored during the reaction time (100 h) as given in Fig. 6. The results demonstrated that the TOC value increased to 7.76 mg/L with time due to the organic intermediates generated because of the degradation of PBs. However, the TOC started to decrease after 10 h and reached 1 mg/L after 100 h due to the attack of the produced radicals on the organic by-products. Full mineralization could not be achieved due to the frequent generation of organic intermediates as a result of the degradation of polymeric chain in PBs.

A comparison between the results in our study and the results in the previous studies is provided in Table 5. Based on the comparison factor, the degradation performance in our study is dazzling, and the synthesized catalyst is efficient, as it can achieve high mass loss ratio under the optimal conditions in the case of polyethylene plastics (1 cm × 1 cm) compared to lower mass loss ratio in other studies.

**Table 5:** Comparison with the results in the previous studies

Catalyst	Plastic characteristics	Operating conditions	Degradation performance	Comparison factor (h^−1^)	References
Boron-goethite	Polyethene plastic	Light source = metal halide lamp (250 W), reaction time = 300 h, catalyst dose = 1 g/L, and plastic concentration = 0.40 g/L	Mass loss ratio = 9.5%	0.01	(Liu et al. 2010)
Plasmonic platinum/zinc oxide nanorod	Low-density polyethylene (LDPE) (1 cm × 1 cm)	Light source = Halogen lamp (50 W), light intensity = 60–70 Klux, and reaction time = 175 h	Carbonyl index = 1.49	−	(Zinc et al. 2019)
NiAl_2_O_4_	Low-density polyethylene (LDPE) (3 cm × 3 cm)	Light source = metal halide lamp (350 W), reaction time = 5 h, and dimethyl sulfoxide (solvent of plastic to accelerate the degradation) = 20 mL	Weight loss ratio = 12.50%	−	(Venkataramana et al. 2021)
N–TiO_2_	High-density polyethylene (HDPE) and low-density polyethylene LDPE (3 mm × 3 mm and 5 mm × 5 mm)	Light source = LED lamp (50 W, 400–800 nm), plastic concentration = 0.40 g/L, catalyst dose = 4 g/L, pH = 3, and reaction time = 50 h	Mass loss ratios = 0.22%, 1.38%, and 0.97% in the case of HDPE, LDPE (3 mm × 3 mm), and LDPE (5 mm × 5 mm), respectively	0.0004, 0.0027, and 0.00194 for HDPE, LDPE (3 mm × 3 mm), and LDPE (5 mm × 5 mm), respectively	(Llorente-García et al. 2020)
Boron-doped cryptomelane	Polyethylene (PE) film (5 cm × 5 cm)	Light source = ultraviolet lamp (20 W, 254 nm), reaction time = 288 h, catalyst dose = 2.50 g/L, plastic concentration = 0.30 g/L, and temperature = 25 °C	Mass loss ratio = 19.90%	0.008	(Liu et al. 2012)
Fe –Ag– TiO_2_	Polyethylene (PE) film (radius 4 cm)	Light source = two UV lamp (6W), light intensity = 1.40 mW/cm^2^, catalyst dose = 0.50 g/L, plastic concentration = 0.10 g/L, and reaction time = 300 h	Mass loss ratio = 14.34%	0.009	(Asghar et al. 2011)
S–TiO_2_	Low-density polyethylene (LDPE) (1 cm × 1 cm)	Light source = metal halide lamp (400 W), PBs concentration = 0.10 g/L, photocatalyst concentration = 3 g/L, pH = 3, reaction time = 100 h, and temperature = 40 °C	Mass loss ratio = 21.74%	0.007	This study

### Degradation mechanism

The illumination of S-TiO_2_ by the metal-halide lamp could generate electrons and holes. Text S1 shows the potential of valence and conduction bands. According to the valence and conduction bands positions, electrons in the conduction band (CB) and holes in the valence band (VB) could generate superoxide radicals and hydroxyl radicals, respectively as a result of the reaction with oxygen and hydroxyl ions, respectively. The sulfur ions doped into the lattice structure of TiO_2_ could trap the excited electrons which enhanced the charge carriers separation as affirmed by the PL spectra in Fig. S2. The generated ^•^OH could attack on the weak places in the polymeric chain generating polyethylene alkyl radicals (Eq. (5)) (Tofa et al. 2019a). Then, peroxy radicals could be produced via reacting the alkyl radicals with oxygen as shown in Eq. (6). Subsequently, peroxy radicals could abstract hydrogen from another polymeric chain to generate hydroperoxide species as described in Eq. (7). The generated hydroperoxide could be converted to two radicals (oxy and hydroxyl radicals) due to the cleavage of the weak O–O bond as given in Eq. (8). Oxy and hydroxyl radicals could abstract hydrogens from other polymeric chains as shown in Eq. (9) which accelerated the degradation of PBs leading to the alteration of its molecular weight. All the generated radicals could depolymerize PBs to monomers and finally would be mineralized to CO_2_ and H_2_O.

5 $${\left(-{{\text{CH}}}_{2}{{\text{CH}}}_{2}-\right)}_{{\text{n}}} {+}^{\bullet }{\text{OH}}\to {\left({-}^{\bullet }{{\text{CHCH}}}_{2}-\right)}_{{\text{n}}}+{{\text{H}}}_{2}{\text{O}}$$

6 $${\left({-}^{\bullet }{{\text{CHCH}}}_{2}-\right)}_{{\text{n}}}+{{\text{O}}}_{2}\to {\left(-{{\text{CH}}}_{2}-{{\text{HCOO}}}^{\bullet }-{{\text{CH}}}_{2}\right)}_{{\text{n}}}$$

7 $${\left(-{{\text{CH}}}_{2}-{{\text{HCOO}}}^{\bullet }-{{\text{CH}}}_{2}\right)}_{{\text{n}}}+{\left(-{{\text{CH}}}_{2}{{\text{CH}}}_{2}-\right)}_{{\text{n}}} \to {\left(-{{\text{CH}}}_{2}-{\text{HCOOH}}-{{\text{CH}}}_{2}-\right)}_{{\text{n}}}+{\left({-}^{\bullet }{{\text{CHCH}}}_{2}-\right)}_{{\text{n}}}$$

8 $${\left(-{{\text{CH}}}_{2}-{\text{HCOOH}}-{{\text{CH}}}_{2}-\right)}_{{\text{n}}}\to {\left(-{{\text{CH}}}_{2}-{{\text{HCO}}}^{\bullet }-{{\text{CH}}}_{2}-\right)}_{{\text{n}}}{+}^{\bullet }{\text{OH}}$$

9 $${\left(-{{\text{CH}}}_{2}-{{\text{HCO}}}^{\bullet }-{{\text{CH}}}_{2}-\right)}_{{\text{n}}}+{\left(-{{\text{CH}}}_{2}{{\text{CH}}}_{2}-\right)}_{{\text{n}}}\to {\left({-}^{\bullet }{{\text{CHCH}}}_{2}-\right)}_{{\text{n}}}+{\left(-{{\text{CH}}}_{2}-{\text{HCOH}}-{{\text{CH}}}_{2}-\right)}_{{\text{n}}}$$

**Fig. 7:**
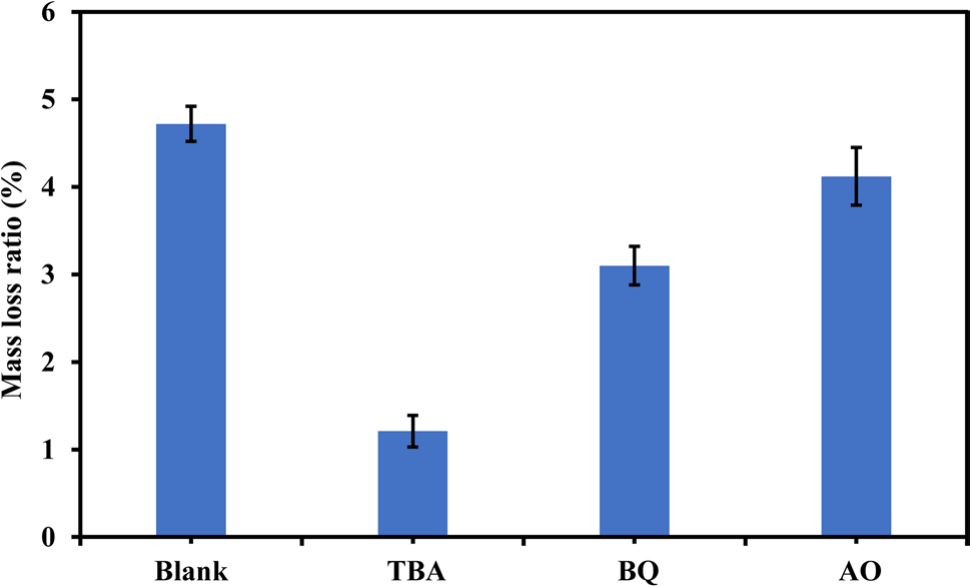
Trapping experiments for specifying the major reactive species

The primary radicals were specified using quenching experiments. Tert-butyl alcohol (TBA), benzoquinone (BQ), and ammonium oxalate (AO) were used for quenching hydroxyl radicals, superoxide radicals, and holes, respectively as shown in Fig. 7 at reaction time of 7 h, pH 3, catalyst dosage of 3 g/L, and PBs concentration of 0.10 g/L. The mass loss ratio decreased to 1.21%, 3.10%, and 4.12% in the case of TBA, BQ, and AO, respectively compared to 4.72% in the absence of quenchers (Blank) affirming the vital contribution of hydroxyl radicals to degrading PBs. This also ensures that superoxide radicals and holes slightly participate in the degradation process.

### Recyclability of S-TiO_2_

**Fig. 8:**
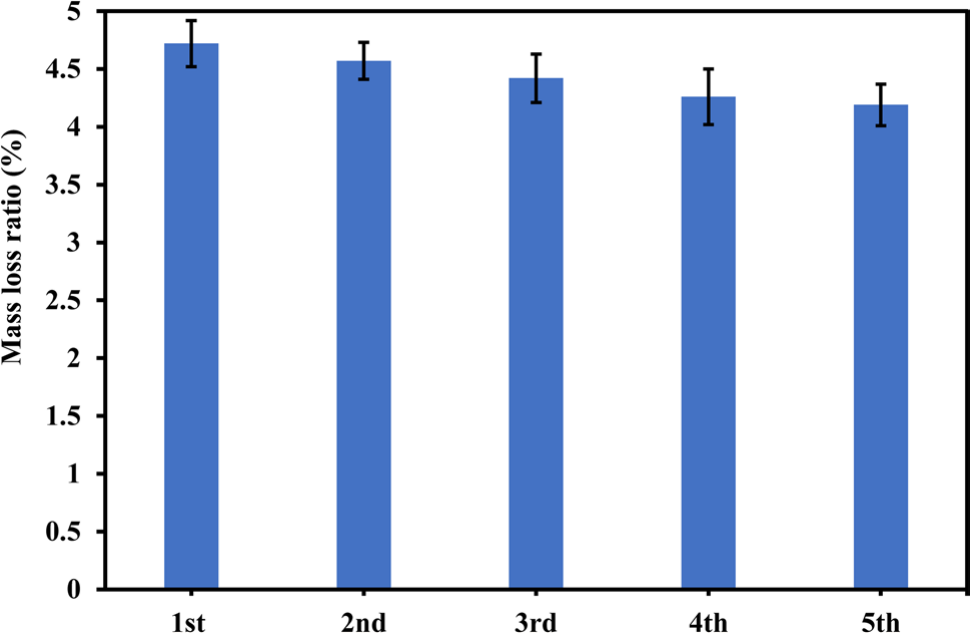
Reusability of the prepared catalyst

The recyclability of the catalyst was examined under five cycles (Time of each cycle = 7 h) as depicted in Fig. 8 at pH 3, catalyst dosage of 3 g/L, and PBs concentration of 0.10 g/L. The catalyst was gathered after each cycle and dried before the next cycle. The mass loss ratios were 4.72%, 4.57%, 4.42%, 4.36%, and 4.19% in the five subsequent runs. The decline in mass loss ratio in successive cycles could be owing to the loss of the catalyst, as some particles could be adsorbed on the PBs surface in each cycle leading to the decrease of catalyst dose (Venkataramana et al. 2021). Further, the generated by-products could cover the active sites of the catalyst in successive cycles which decreased the generated radicals (Samy et al. 2021b).

## Conclusions

The synthesized photocatalyst’s bandgap is 2.85 eV indicating the ability of S-TiO_2_ to generate radicals under visible light radiation. Using 1.25 g/L of the catalyst and 0.10 g/L of PBs at pH 5 and reaction time of 7 h attained a mass loss ratio of 2.70%. To improve the weight loss ratios, the degradation of PBs was also examined under pH 2 and 3 showing that the weight loss ratio increased to 3.10% in the case of pH 3 compared to 2.90% in the case of pH 2. Additionally, the increase of catalyst dose to 3 g/L and 5 g/L raised the mass loss ratios to 4.72% and 4.89%, respectively. Further, prolonging the reaction time to 100 h at pH 3, catalyst dose of 3 g/L, and PBs concentration of 0.10 g/L resulted in attaining a mass loss ratio of 21.74%. The presence of cracks on PBs surface after photodegradation for 100 h as depicted in the SEM micrograph and the increase of carbonyl index from 0.52 in the case of pristine PBs to 1.41 in the case of degraded PBs assured the photodegradation of the polymeric chain in PBs. TOC analysis affirmed the degradation of PBs to organic intermediates and the ability of the generated reactive species to mineralize the organic by-products. Hydroxyl radicals were the dominant reactive species, and the synthesized catalyst could be reused efficiently for five runs. The prepared catalyst proves its effectiveness and stability towards the degradation of PBs. Therefore, it can be employed in a large-scale system to efficiently degrade plastics.

### Supplementary Information

Below is the link to the electronic supplementary material.

Supplementary file1 (DOCX 184 KB)

## Data Availability

All data generated or analyzed during this study are included in this published article [and its supplementary information files].
