# Supplementary material for: Photodegradation of polyethylene debris in water by sulfur-doped TiO2: system optimization, degradation mechanism, and reusability
Source: Environ Sci Pollut Res Int. 2023 Dec 14;31(3):3951–63. doi: 10.1007/s11356-023-31460-1 (PMC10794281; doi:10.1007/s11356-023-31460-1)
Supplement: Supplementary file 1 — Supplementary file1 (DOCX 184 KB) [file 11356_2023_31460_MOESM1_ESM.docx]

**Photodegradation of polyethylene debris in water by sulfur-doped TiO_2_: System optimization, degradation mechanism, and reusability**

Ahmed Sharara ^1^, Mahmoud Samy ^[[1]](#footnote-1), *^, Mohamed Mossad ^1^, Mohamed Gar Alalm ^1^

^1^ *Public Works Engineering Department, Faculty of Engineering, Mansoura University, Mansoura, 35516, Egypt*


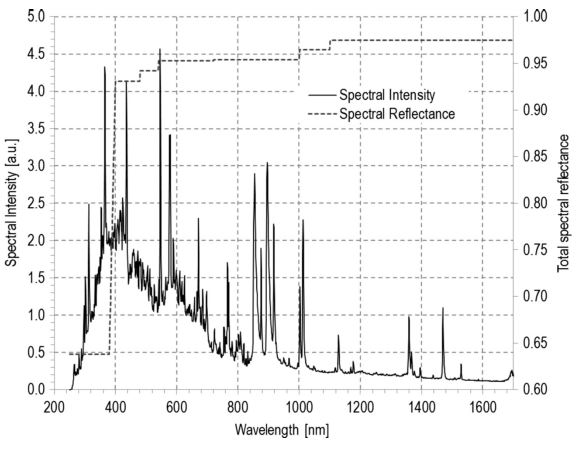


**Fig. S1.** Emission profile for a typical metal halide lamp (Roba and Siegel 2017).


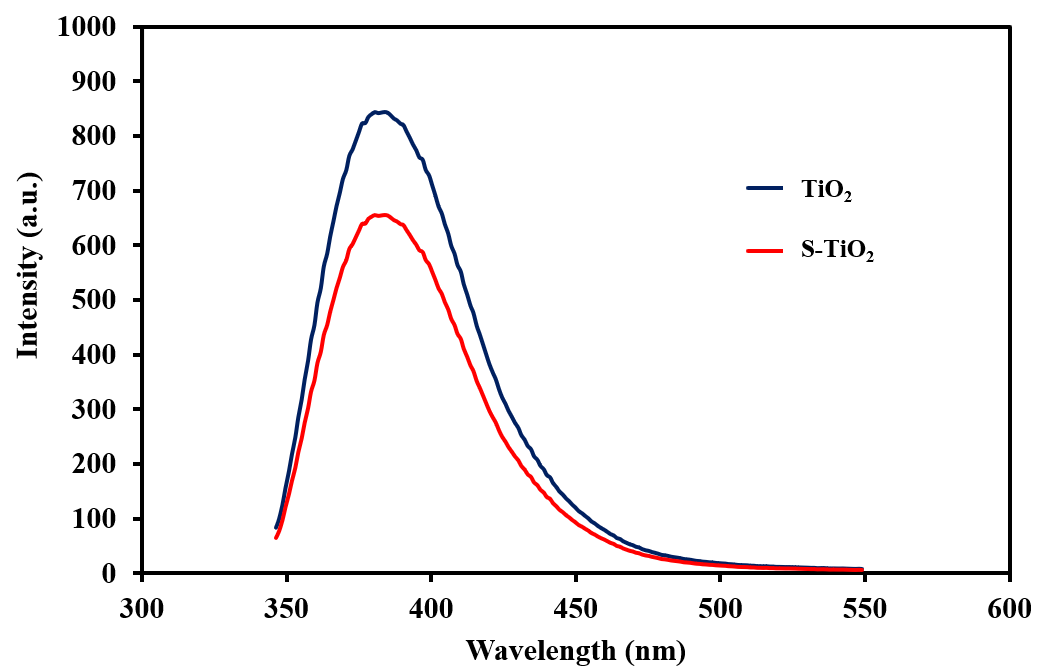


**Fig. S2.** PL spectra of TiO_2_ and S-TiO_2_.

**Fig. S3.** Effect of light intensity on the mass loss ratios.

**Text S1:**

The valence and conduction band positions can be estimated according to Eqs. (1, 2) (Rahimi et al. 2019):

E_CB_ = X - E_C_ - 0.5E_g_  (1)

E_VB_ = E_CB_ + E_g_ (2)

where E_CB_ and E_VB_ are the potentials of conduction and valence bands, respectively, X is the absolute electronegativity (5.58 eV for S-TiO_2_), E_g_ is the bandgap of the catalyst, and E_C_ is the free electron energy of hydrogen scale (~4.50 eV) (Grinnell and Samokhvalov 2018). According to the above equations, the potentials of valence and conduction bands are 2.505 eV and -0.345 eV, respectively. The conduction band potential of S-TiO_2_ is higher than the reduction potential of O_2_/^-^O_2_• (-0.33 eV) and the valence band potential of S-TiO_2_ is higher than the oxidation potential of OH^-^/•OH (2.40 eV) which could result in the production of ^-^O_2_• and •OH from the reduction of O_2_ and the oxidation of OH^-^, respectively (Wang et al. 2023).

**References**

Grinnell C, Samokhvalov A (2018) Exploring the electronic structure of aluminum metal-organic framework Basolite A100: Solid-state synchronous fluorescence spectroscopy reveals new charge excitation/relaxation pathways. Phys Chem Chem Phys 20:26947–26956. https://doi.org/10.1039/c8cp04988b

Rahimi B, Jafari N, Abdolahnejad A, et al (2019) Application of efficient photocatalytic process using a novel BiVO/TiO_2_-NaY zeolite composite for removal of acid orange 10 dye in aqueous solutions: Modeling by response surface methodology (RSM). J Environ Chem Eng 7:103253. https://doi.org/10.1016/j.jece.2019.103253

Roba JP, Siegel NP (2017) The design of metal halide-based high flux solar simulators: Optical model development and empirical validation. Sol Energy 157:818–826. https://doi.org/10.1016/j.solener.2017.08.072

Wang H, Yu S, Gao T, et al (2023) The efficient degradation of organic pollutants by Z-scheme MIL-88A@TiO_2_ heterojunction photo-Fenton catalyst: The synergistic effect of photocatalysis and Fenton catalysis. J Alloys Compd 960:170688. https://doi.org/10.1016/j.jallcom.2023.170688

1. * Corresponding authors:

   Tel: +201018262849. E-mail address: [mahmoud.samy@ejust.edu.eg](mailto:mahmoud.samy@ejust.edu.eg); [msa203050@mans.edu.eg](mailto:msa203050@mans.edu.eg) (Mahmoud Samy) [↑](#footnote-ref-1)
